# Home-based preoperative rehabilitation (prehab) to improve physical function and reduce hospital length of stay for frail patients undergoing coronary artery bypass graft and valve surgery

**DOI:** 10.1186/s13019-017-0655-8

**Published:** 2017-10-26

**Authors:** Iain Waite, Ranjit Deshpande, Max Baghai, Tania Massey, Olaf Wendler, Sharlene Greenwood

**Affiliations:** 1 Department of Physiotherapy, Kings College Hospital NHS Trust, London, England; 2 Department of Cardiology, Kings College Hospital NHS Trust, London, England; 3 Department of Physiotherapy and Renal Medicine, Kings College Hospital, London, England; 4 Renal Medicine, Division of Transplantation Immunology & Mucosal Biology, King’s College London, London, England

**Keywords:** Cardiac surgery, Rehabilitation, Prehab, Frailty, Length of stay

## Abstract

**Background:**

Evidence suggests that elective cardiac patients are at risk of functional and psychological deterioration in the time preceding surgery. This poses a risk to successful post-operative rehabilitation. This prospective one-group pre-test, post-test evaluation was designed to assess a clinical Pre-operative Rehabilitation (PREHAB) home-based exercise programme, to optimise pre-operative physical function and frailty in patients awaiting elective Coronary Artery By-Pass Graft (CABG) or Valve Surgery.

**Method:**

Consenting patients awaiting cardiac surgery, with wait time ≥ 6 weeks were referred to a Senior Physiotherapist for baseline assessment. Patients were offered PREHAB in the form of functional home-based exercise that was prescribed from baseline physical outcomes. All patients were followed up via telephone to ensure progression of exercise and any problems associated with it. This continued weekly until the patient attended Surgical Pre-assessment clinic, where all outcome measures were re-assessed.

**Results:**

Twenty two patients, out of a total number of 36 patients seen in the surgical clinic between March 2016 and August 2016, participated in the prehab clinical evaluation. Twenty patients completed their prescribed exercises on a weekly basis prior to surgery. No adverse events or cardiac symptoms were reported as a result of the home exercise intervention. Paired t-Test analyses revealed a significant mean difference in clinical frailty score (CFS) of −0.53 ± 0.51 (95% CI [−0.774, −0.279], *P* = 0.0003). Significant mean difference in six-minute walk test (6MWT) distance of 42.5 ± 27.8 m (95% CI [23.840, 61.251], *P* = 0.0005), 6MWT walking speed of 0.5 ± 0.4kmh (95% CI, [0.2433, 0.7567], *P* = 0.001), and short physical performance battery (SPPB) total score of 2.2 ± 1.7, (95% CI [3.066, 1.200], *P* = 0.0002) were also observed. The change in 6MWT distance was shown to be significantly associated with hospital length of stay (LOS) (*r* = 0.7; *P* = 0.03).

**Conclusion:**

This small exploratory evaluation suggests that providing a home-based PREHAB programme for frail patients undergoing CABG or Valve surgery may be able to improve functional ability and reduce hospital length of stay for those patients undergoing cardiac surgery. A frailty score with greater sensitivity may be required to elucidate the influence frailty could have in reducing length of stay. A large randomised controlled study is required to reveal the potential beneficial effects of PREHAB in this patient population.

## Background

By the year 2039, it is estimated that 24.3% of the UK population will be aged 65 or over. Advancements in technology, medical treatment and improved post-surgical morbidity and mortality, means the number of older adults undergoing surgical procedures is increasing faster than the rate of the population aging [1–4]. This aging population present with greater comorbidities and frailty, in turn posing significant risk to a successful post-operative recovery. Wait times for elective cardiac surgery vary but evidence shows patients ultimately deteriorate functionally and psychologically during this period prior to surgery.

The use of frailty as a risk factor or determinant in predicting adverse postsurgical outcome is evolving [5, 6]. Longer wait times for surgery see a trend towards deterioration in both a patients’ psychological and physiological condition, suggesting that the overall condition of those awaiting cardiac surgery should be monitored continuously [7]. It’s indicated that as much as 45% of patients claimed their own health had suffered along with raised levels of anxiety amongst spouses [8].

Older adults undergo surgery twice as often as their younger counterparts [9], and commonly have a lower functional reserve in many body systems, making it more difficult to atone for the stress of surgery. Consequently, they often face greater postoperative complications. [9, 10]. Defining and categorising frailty has long created a challenge for health care professionals and researchers. Frailty can be described as a state of risk or vulnerability, a precarious balance between demands and capacity to cope and impending or current disability [11]. Frailty is considered by some to exist independently with its own pathophysiology [12], but others describe it as accelerated aging or an accumulation of age related deficits [13]. Symptoms of frailty may be present in any age group [14, 15], although are perhaps more prevalent in the older adult that is exposed to a natural decline in physical and psychological function.

The advance in modern medicine is a major contributory factor to people living longer, and an overall increase in the elderly population. This increasing number of older adults, combined with the fact that older age brings augmented comorbidities, will inevitably lead to a greater incidence of frailty and subsequent heightened surgical risk. Stresses to the patient undergoing cardiac surgery are well documented and despite ever improving medical care, post-operative medical complications are more frequent in the older population. These include increased hospital length of stay (LOS), decreased physical function and decreased health related quality of life (HRQOL). There is a need for alternative or additional approaches, for optimising physical function and post-operative recovery. Preoperative rehabilitation is a concept which has been extensively studied within the orthopaedic population and has demonstrated significant reduction in the need for post-operative rehabilitation [16]. Meta-analyses of studies investigating pre-operative exercise in patients with lung cancer shows significant increase in pulmonary function with positive increases in exercise capacity [17]. Currently, within the cardiac population there is limited research about the use of preoperative rehabilitation. The studies typically concentrate on cardiopulmonary outcomes and the utilisation of respiratory physical therapy as an intervention. These interventions have resulted in some reduction in post-operative complications, and decreased hospital LOS [18]. A study by Arthur et al., [19] investigated the use of group exercise in patients awaiting CABG and demonstrated a significant decrease in hospital length of stay, increases in health related quality of life and improved recruitment to cardiac rehabilitation. These studies included patients that were physically able to travel and attend group exercise sessions, an intervention that frail patients, who are awaiting cardiac surgery would find difficult to engage with. A recent meta-analysis conducted by Jolly et al., has shown that home-based post-operative cardiac rehabilitation is as effective as hospital-based rehabilitation [20]. To date, no studies have evaluated the use of home-based pre-operative exercise rehabilitation for frail patients awaiting cardiac surgery.

The early preoperative assessment is a fundamental exchange of information between health care professionals, patients and their families. It provides an opportunity for both sides to evaluate the level of surgical risk, discuss prospective surgical and patient-based outcomes. Early preoperative assessment may also be an ideal opportunity to identify modifiable risk factors such as frailty. The time spent waiting for cardiac surgery to be scheduled, is a period of great uncertainty for a patient. There appears to be heightened anxiety regarding the inclusion of physical activity in this time period, mainly as a result of their current cardiac condition or diagnosis [21]. A lack of engagement with any physical activity or planned exercise training, coupled with increasing levels of sedentary behaviour, can lead to physical deconditioning and an increased level of frailty. The timely identification of an increased level of frailty at the early preoperative assessment, may provide a unique opportunity to engage patients with a tailored intervention during the surgery wait time (typically 6 weeks), enabling a preoperative optimisation of functional ability, confidence, motivation and a reduced probability of post-operative complications. This exploratory clinical intervention was designed as a clinical pilot study to assess a clinical Pre-operative Rehabilitation (PREHAB) home-based exercise programme, to optimise pre-operative physical function and frailty in patients awaiting elective Coronary Artery By-Pass Graft (CABG) or Valve surgery.

## Methods

### Study design

A prospective, single-centre exploratory clinical pilot designed to assess and treat patients identified as frail who have been listed for elective Cardiac Artery Bypass Graft (CABG) or Valve Surgery (VS). The pilot study aimed to explore the use of a pre-surgical rehabilitation ‘PREHAB’ intervention, in the form of home-based exercise therapy, with the intention to optimise pre-surgical physical function.

### Patients

Patients seen in early preoperative assessment clinics were voluntarily enrolled into the prehab programme when listed for cardiac surgery. The inclusion criteria for the programme included those aged ≥65 years, undergoing elective Surgery for CABG, Valve repair/replacement (via sternotomy), Transcatheter Aortic Valve Implantation (TAVI) and patients with an estimated ≥6 week surgical waiting list time. Participants were excluded if they required emergent surgery (Surgery for which there should be no delay due to ongoing refractory cardiac compromise), clinical instability, decompensated heart failure not yet stabilised, and any acute process causing significant symptoms or abnormal vital signs.

### Outcomes and assessments.

**Fig. 1:**
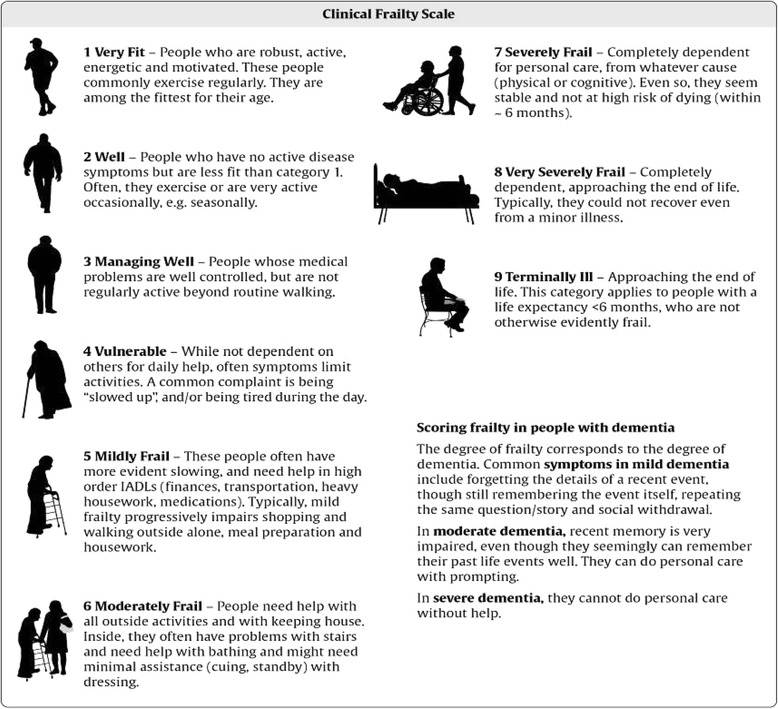
Clinical Frailty Score: Canadian study on health and aging revised 2008 [22]

The main aim of this clinical pilot was to explore the potential for creating a PREHAB intervention for those patients who were frail, and awaiting cardiac surgery. Patient post-surgical length of stay (LOS) was measured by the number of days from their date of surgery to the date of their discharge home from hospital. Functional capacity was assessed using 3 independent measures including, the Duke Activity Status Index (DASI) [22], the Short Physical Performance Battery Protocol (SPPB) [23] and the 6 min walk test (6MWT) [24]. The DASI is a self-administered questionnaire that measures a patient’s functional capacity. The SPPB is a test designed to measure a patient’s functional status and physical performance and consists of a 3-stage balance assessment, a walking speed measure and an assessment of lower limb functional strength. The 6MWT is a submaximal exercise test to measure cardiorespiratory fitness and functional capacity. Heart rate and oxygen saturation was monitored throughout the 6MWT. Patient anxiety and depression levels were scored with the Hospital anxiety and depression scale (HADS) [25]. Body mass index (BMI) was measured in line with the formula weight (kg) / height^2^ (m^2^). Patient Frailty status was assessed using the clinical frailty scale (CFS) with its 9 point measure ‘very fit’ to ‘terminally ill’ as seen in Fig. 1, [26]. All outcome measures were assessed at baseline, and again at pre-assessment clinic. Patient agreement to engage with a post-surgical cardiac rehabilitation programme was also recorded. An anonymised patient satisfaction questionnaire was provided for patients to evaluate their experience and understanding of the PREHAB intervention.

### ‘Prehab’ intervention

Following completion of all baseline outcome measures, a prehab exercise intervention was individually tailored by a specialist physiotherapist for each patient. All exercises were demonstrated and observed in the hospital setting, prior to patients initiating their home programme. The exercise prescription was adapted for patients of differing physical abilities and needs. Balance and strength-training exercises, with progressive levels were modelled and developed from the ‘Otago exercise programme to prevent falls in older people’ [27]. This is shown in Table 1 (balance exercise prescription) and Table 2 (Strength exercise prescription). Patients were encouraged to self-monitor their levels of exercise intensity using the rating of perceived exertion scale (RPE) [28] and were given an individual level at which to exercise, which was dependent on their current cardiac status, symptoms and comorbidities. Progression of strength-training exercises was determined by patient reported reduction in RPE level for ≥1 week or ≥3 sessions without any exacerbation of cardiac symptoms. Balance exercise levels were progressed based on patients self-reported ability to safely complete those at their current level. Patients were encouraged to keep a home exercise diary and report any problems, such as pain or discomfort, so that they could be appropriately advised with any necessary exercise alterations instigated. During telephone consultations, patients would be encouraged, and commended on their efforts.

**Table 1:** Outline of the home based balance exercises given (based on Otago exercise programme balance exercises for fall prevention) [13], including levels of progression from (A) – simplest to (D) – most challenging

Balance Exercise				
	Level A	Level B	Level C	Level D
Knee Bends	10 repetitions Hold support	10 repetitions No support	10 repetitions repeat 2 times No support	10 repetitions, repeat 3 times No support
Backwards Walking	----------	10 steps, 4 times Hold support		10 steps, 4 times No support
Walking And Turning Around	----------	Walk and turn around (do a figure 8) twice Use walking aid	Walk and turn around (do a figure 8) twice No support	----------
Sideways Walking	----------	10 steps, 4 times Use walking aid	10 steps, 4 times No support	----------
Tandem Stance (Heel Toe Stand)	10 s Hold support	10 s No support		----------
Tandem Walk (Heel Toe Walk)	----------	----------	Walk 10 steps Hold support, repeat	Walk 10 steps No support, repeat
One Leg Stand	----------	10 s, Hold support	10 s, No hold	30 s, No hold
Heel Walking	----------		10 steps, 4 times Hold support	10 steps, 4 times No support
Toe Walk	----------	----------	10 steps, 4 times Hold support	10 steps, 4 times No support
Heel Toe Walking Backwards	----------	----------	----------	Walk 10 steps No support, repeat
Sit To Stand	5 stands, 2 hands for support	5 stands, 1 hand or 10 stands, 2 hands for support	10 stands, no support *or* 10 stands, 1 hand for support, repeat	10 stands No support, repeat
Stair Walking	As instructed	As instructed	As instructed	As instructed

**Table 2:** Outline of the home based strength exercises given, including levels of progression from (A) – simplest through to (D) – most challenging

Strength Exercise				
	Level A	Level B	Level C	Level D
Mid Row	Yellow Band	Red Band	Blue Band	Blue Band (single arm)
Wall Push Up	No Band	No Band	No Band	No Band
Heel Raises	No Band	No Band	No Band	No Band
Sit to Stand	Use arms to assist standing	Arms straight out in front	Arms across Chest	Arms across Chest
Leg Abduction	No Band	No Band	Blue Band	Blue Band
Bicep Curl	Yellow Band	Red Band	Blue band	Blue band (Single arm)
Seated Leg Extension	No Band	No Band	No Band	No Band

### Statistical analysis

SPSS version 20 was utilised for all analyses. Descriptive statistics, comprising of mean (SD), were used to define baseline and post intervention results. Paired sample t-Tests were performed to examine pre and post-test differences in CFS, weight, BMI, HADS, DASI, SPPB and 6MWT. Pearson’s rank correlation was used to examine associations between frailty, functional capacity and hospital LOS.

## Results

Twenty two patients, out of a total number of 36 patients seen in the early pre-operative assessment clinic between March 2016 and August 2016, participated in the prehab clinical pilot study. 90% of patients (*n* = 20) completed their prescribed exercises 3×/week prior to surgery, with a total of 15 patients attending the surgical pre-assessment appointment prior to surgery. No adverse events or cardiac symptoms were reported as a result of the home exercise intervention. The pre-post intervention results are shown in Table 3.

**Table 3:** Pre-Post intervention results for patients completing the PREHAB programme

	PD (*n* =)	*t* (df), *P*	% improvement
	Mean (SD)		
CFS	20		
Pre	4.58 (.961)		
Post	4.05 (1.177)	4.472 (18), <0.001	11.6%
Weight	20		
Pre	76.05 (15.67)		
Post	76.30 (15.39)	−0.295 (19), 0.772	−0.3%
BMI	20		
Pre	26.10 (3.57)		
Post	26.18 (3.34)	−0.250 (19), 0.805	−0.01%
Anxiety	15		
Pre	6.47 (4.88)		
Post	6.33 (4.70)	0.274 (14), 0.788	2.7%
Depression	15		
Pre	4.73 (3.97)		
Post	4.33 (3.85)	1.193 (14), 0.253	8.5%
SPPB (BAL)	15		
Pre	2.87 (1.302)		
Post	3.73 (.458)	−3.166 (14), 0 .007	30%
SPPB (GAIT)	15		
Pre	2.80 (1.082)		
Post	3.27 (.799)	−2.432 (14), 0.029	16.8%
SPPB (CHAIR)	15		
Pre	1.40 (1.352)		
Post	2.27 (.961)	−4.026 (14), 0.001	30%
SPPB (TOTAL)	15		
Pre	7.13 (3.021)		
Post	9.27 (1.624)	4.904 (14), <0.001	30%
DASI	15		
Pre	25.19 (3.105)		
Post	25.66 (2.869)	−0.437 (14), 0.669	1.9%
6MWT(Distance)	11		
Pre	236.64 (146.66)		
Post	279.18 (126.95)	5.068 (10), <0.001	18%
6MWT (Speed)	11		
Pre	2.291 (1.420)		
Post	2.791 (1.268)	4.340 (10), 0.001	21.8%

Paired t-Test analyses revealed a significant mean difference in CFS of −0.53 ± 0.51 (95% CI [−0.774, −0.279], *P* = 0.0003). Significant mean difference in 6MWT distance of 42.5 ± 27.8 m (95% CI [23.840, 61.251], *P* = 0.0005), 6MWT walking speed of 0.5 ± 0.4kmh (95% CI, [0.2433, 0.7567], *P* = 0.001), and SPPB total score of 2.2 ± 1.7, (95% CI [3.066, 1.200], *P* = 0.0002) were also observed. The change in 6MWT distance was shown to be significantly associated with hospital LOS (*r* = 0.7; *P* = 0.03). Patients improved their physical function (SPPB 30%, 6MWT 18%) and showed reduction in the CFS (18%). Hospital LOS was not shown to be significantly associated with a reduction in CFS. Despite an increase in physical function, there was no significant reduction in BMI. BMI was able to be maintained in the time period (0.3% increase in mean body weight). Mean scores of anxiety and depression showed a non-significant improvement of 2.7% & 8.5% following the intervention. Mean hospital LOS was 8.9 days. Three patients in the sample endured a prolonged LOS due to unforeseen surgical complications that were unrelated to frailty or physical function. No patient discharges were delayed due to physical or functional ability, and when the 3 patients with unrelated complications were removed from analyses, mean LOS was 5.8 days. 100% of patients were discharged back to their own home, with only 1 requiring an initial package of care for assistance with domestic tasks. 100% of participants were offered Cardiac Rehabilitation, and there was a subsequent uptake of 82%.

## Discussion

As an exploratory study, great importance was placed on constant re-evaluation, examining the design and procedural elements at regular intervals, considering any required changes in its template and structure. This re-evaluation promptly identified a low number of referrals from the surgical clinic, with further analysis indicating the primary reason for exclusion (55%) to be patients having to be ≥65 years of age. This specific exclusion criteria was set to identify the more frail patients and recruit a manageable quantity of patients. It was subsequently amended to enable frail patients to be referred regardless of age. This change highlighted the fact that the traits of frailty can be prevalent at varying ages [14]. As well as using this time to collect baseline information and prescribe individually specific interventions, patients had the opportunity to gain greater understanding of their pending surgery. The majority of patients referred for surgery reported a physical or psychological decline in the time leading up to their surgical clinic and admitted to having slowed down or reduced the amount of activity they were now doing. The amount or rate of decline was understandably variable from patient to patient. However, in the majority of patients this was a rapid and significant decline leading to changes in their lifestyle. It also brought about forced adaptions in the activities of daily living, for both the patient and those around them, comparable to previous findings [8]. This self-realisation of decline may have been a contributory factor as to why so many of the enrolled patients were willing to follow the exercise intervention so rigorously. Using an anonymous patient feedback questionnaire, 90% of patients recruited to the study reported to complete the rehabilitation exercises ≥3 times per week. Similar research has reported both positive [29] and inconsistent [19] levels of adherence, however there are no previous comparable studies in a frail population using an independent home based exercise as their intervention. This study demonstrated that significant improvements in functional ability and exercise capacity were achievable in those patients who completed the home-based Prehab programme, although patients’ overall perceived ability, measured with the DASI, only demonstrated a minimal improvement. A single patient was identified to have anxiety and depression scores with the need for referral to clinical Psychology. Whilst there was no statistically significant change in HADS score post-intervention within this small sample, it is perhaps understandable that anxiety would remain high within a cohort of patients awaiting cardiac surgery, and this has certainly been seen in previous studies [19, 30]. Whilst this study demonstrates that it is feasible to implement a home-based exercise intervention within a clinical real-world setting, a randomised controlled trial is warranted to assess the effect of this novel intervention. This exploratory study has several limitations. Adherence to the exercise intervention was self-reported by participants and there was a varying intervention time that was dependent on wait times between early pre-operative clinic and surgery.

## Conclusion

This small exploratory study suggests that providing a real-world clinical home-based PREHAB programme for frail patients undergoing CABG or Valve surgery is feasible, and has the potential to improve functional ability and reduce hospital length of stay for those patients undergoing cardiac surgery. A frailty score with greater sensitivity may be required to elucidate the influence frailty could have in reducing length of stay. A large randomised controlled study is required to reveal the potential beneficial effects of PREHAB in this patient population.

## Abbreviations

6MWD: 6 min walk distance
6MWT: 6 min walk test
BMI: Body mass index
CABG: Coronary Artery By-Pass Graft
CFS: Clinical Frailty Score
DASI: Duke Activity Status Index
HADS: Hospital Anxiety and Depression Scale
HRQOL: Health related quality of life
IPAQ: International Physical Activity Questionnaire
LOS: Length Of Stay
PREHAB: Pre-operative Rehabilitation
RPE: Rating of Perceived Exertion
SPPB: Short Physical Performance Battery Protocol
TAVI: Trans catheter Aortic Valve Implantation
VS: Valve Surgery
